# American Medical Association

**Published:** 1857-07

**Authors:** 


### SELECTIONS.

### *American Medical Association.*

TENTH ANNUAL SESSION.

NASHVILLE, May 5, 1857.

The Association met at 11 o'clock, in the Representative Hall of the State Capitol, the President, Dr. Zina Pitcher, of Michigan in the chair, and upon his right Dr. W. K. Bowling of Tennessee, one of the Vice-Presidents. Dr. Wm. Brodie of Michigan, and Dr. R. C. Foster of this city, Secretaries, were present.

The meeting having been duly organized, the first business in order was stated by the Chair to be the reception of the report of the Committee of Arrangements.

Dr. C. K. Winston, chairman of the Committee of Arrangements, on behalf of the committee and of the medical profession of the city generally, extended a sincere and cordial welcome to the members of the Association, in a few pertinent and appropriate remarks. [Published in last number.]

Dr. Winston then proposed that the roll of delegates, who had registered their names, should be read. The roll having been called, it appeared that twenty States were represented.

Upon the suggestion of Dr. C. K. Winston, our venerable fellow citizens, Drs. Felix Robertson, John Shelby, and James Overton were made permanent members of the Association.

The following list comprises the names of all delegates, permanent members, and members by invitation, in attendance during the session:

*Connecticut.*—Charles Hooker.

*New Hampshire.*—Adoniram Smalley.

*New York.*—James R. Wood, D. M. Reese, George N. Burwell, Alden March, Samuel St. John.

*New Jersey.*—Richard M. Cooper.

*Pennsylvania.*—R. Dunglison, B. F. Schneck, Casper Wister, P. Cassidy.

*Georgia.*—Henry F. Campbell, C. R. Walton, N. F. Powers, A. Means, Joseph P. Logan, M. H. Oliver, Thomas S. Powell, J. Gordon Howard, R. D. Arnold, George P. Paddleford, Pike Brown, Jesse Boring.

*Alabama.*—G. M. Merriweather, W. P. Reese, A. F. Alexander, S. W. Clanton, W. H. Thornton, P. C. Winn, T. Stith Malone, W. J. Bass, G. D. Norris, J. F. Sowell, J. W. Morris.

*Tennessee.*—Frank A. Ramsey, James Rodgers, R. O. Currey, B. B. Lenoir, J. L. C. Johnston, J. M. Boyd, George R. Grant, T. A. Atchison, S. S. Mayfield, J. D. Kelly, T. L. Maddin, J. D. Winston, J. E. Manlove, G. A. J. Mayfield, J. D. Kelley, T. L. Maddin, J. D. Winston, J. E. Manlove, G. A. J. Mayfield, Richard Owen, W. P. Jones, J. P. Ford, Robert C. Foster, John H. Callender, John H. Morton, A. H. Buchanan, James W. Hoyte, N. C. Perkins, J. Berrien Lindsley, C. K.

Winston, Paul F. Eve, W. P. Moore, Milo Smith, Wallace Estill, B. W. Avent, H. H. Clayton, H. M. Whitaker, H. B. Malone, T. M. Woodson, A. B. Ewing, Robert Martin, W. K. Bowling, P. S. Woodward, R. F. Evans, Thomas Lipscomb, M. Ransom, J. A. Long, John M. Watson, W. D. Haggard, John S. Park, D. B. Cliff, T. G. Kennedy, T. R. Jennings, Ira Conwell, J. S. Burford, W. H. Childress, W. A. Cheatham, J. F. Town, J. M. Brannock, B. C. Jillson, P. W. Davis, G. F. Smith, W. D. Senter, J. W. McNutt, R. G. P. White, J. P. Epperson, S. L. Wharton, T. C. Murrell.

*Louisiana.*—S. O. Scruggs, Robert A. New, Cornelius Beard, E. D Fenner.

*Kentucky.*—Samuel Annan, R. W. Gaines, J. B. Flint, J. W. Singleton, R. J. Breckinridge, S. C. Porter, W. S. Chipley, S. M. Bemiss, L. G. Ray, W. A. Atchison, E. G. Davis, L. E. Almon, John T. Fleming, C. P. Mattingby, D. W. Yandell.

*Indiana.*—W. H. Byford, W. W. Hitt, Isaac Mendenhall, T. Bullard, N. Johnson.

*Illinois.*—J. C. H. Hobbs, A. H. Luce, James M. Steel, E. K. Crothers, T. K. Edmiston, W. A. Hillis.

*Missouri.*—S. Pollak, E. S. Fraser, John S. Moore, C. A. Pope.

*Michigan.*—A. B. Palmer, L. G. Robinson, Zina Pitcher, W. Brodie, L. H. Cobb, M. Gunn, Lewis Davenport, P. Cline, M. B. Stebbins.

*Iowa.*—Asa Horr, William Watson, D. L. McGugin, J. C. Hughes.

*Ohio.*—Henry F. Koehne, J. Mosgrove, B. S. Brown, D. Ferris, A. W. Munson.

*Wisconsin.*—Hays McKinley, J. K. Bartlett.

*South Carolina.*—E. R. Henderson, M. S. Moore, R. W. Gibbs, R. S. Bailey.

*Mississippi.*—F. B. Shuford, S. S. Cain, J. T. Lowe.

*Arkansas.*—F. Grundy McGavock.

The President then stated that it was customary to take a recess of fifteen minutes in order that the different State Delegations might appoint a member to serve on the Committee on Nominations, and the Association took a recess accordingly for that purpose.

At the expiration of the recess the Association was called to order, and the State Delegations then reported their choice respectively of delegates to serve on the Nominating Committee, which was constituted as follows:

Connecticut, Charles Hooker; New Hampshire, A. Smalley; Indiana, W. W. Hitt; Wisconsin, J. K. Bartlett; New York, James R. Wood; Michigan, A. B. Palmer; Missouri, J. S. Moore; Illinois, T. K. Edmiston; Kentucky, R. J. Breckinridge; Arkansas, F. G. McGavock; Ohio, B. S. Brown; South Carolina, R. W. Gibbes; Alabama, W. P. Reese; Mississippi, F. B. Shuford; New Jersey, R. M. Cooper; Louisiana, S. O. Scruggs; Pennsylvania, P. Cassidy; Georgia, Thomas S. Powell, Tennessee, J. B. Lindsley; Iowa, Asa Horr.

On motion of Dr. Hooker, of Connecticut, it was resolved that the President, Dr. Pitcher, be now requested to deliver his annual address.

*Address of* Zina Pitcher, M. D., *President of the American Medical Association. Delivered on the occasion of the Meeting of the Association in Nashville, May 5th*, 1857.

Assembled as we are here, under the auspices of the medical profession in Tennessee; meeting in presence of the citizens of this beautiful city, honored by representatives from that better part of our creation, who like the Amarnt of Milton, throw their shadows and shed their fragrance o'er the waters of the fount of life; coming as we have in considerable numbers from distant portions of the United States, abandoning for the time being our private engagements, and encountering on our way hither the hazards incident to velocity in locomotion, as if only to enjoy the social amenity and the pleasures of professional re-union, these two questions naturally arise in the minds of those who are merely witnesses of the spectacle presented by our assemblage. "For what purpose is this convocation of physicians? What is there in the nature of their particular pursuit which prompts them thus to relinquish its rewards, to forego the endearments of home, when there is no visible manifestation on their part, of a design to promote those objects which center in self-interest, to advance the purposes of sectarian ambition or political partisanship?"

In the fulfilment of a duty incident to the position which I have had the honor to hold for the past year—a year full of pleasant recollections to myself—I shall, whilst designing in brevity to follow the example of my honored predecessors, attempt an explanation of the phenomenon we may be supposed to present to the mind of an intelligent, but uninitiated observer.

Before entering upon the task I have assigned to myself, I beg you to indulge me one moment, in repeating to the Association my assurances of gratitude for the distinction I have received at its hands, and for the personal manifestations of confidence and the acts of courtesy I have received from many of the individual members, the recollections of which will linger in my memory and lessen the consciousness of my weight of years, on the remainder of my journey down the declivity of life.

The objects for which the Association was formed will ever enlist my warmest sympathies and command my active co-operation. I congratulate you on the happy circumstances by which this anniversary meeting is attended, on the evidences of vigor and the promises of longevity which this Association derives from its annual migrations. God grant that its existence may be prolonged by these annual renewals of its vitality, so long as there are evils for it to reform, or works of beneficence for it to accomplish.

To do what I have proposed satisfactorily to myself, would involve the necessity of showing the relation which medicine has held to the civil authority, to the ecclesiastical power and to the social condition of the people for all time antecedent to this organization. This review would also lead us to consider the relation which free governments bear to letters, to science and the arts; a field too large for us to occupy on the present occasion. We shall endeavor without attempting all this, to present to your view the condition of the profession at the time this organization sprang out of the antecedent chaos, the cause or causes of that condition; whether inherent and incurable, or whether arising from intrinsic circumstances, which may be remedied, and whether this remedy is to be found in public authority, or sought for in associated professional influence.

We remark first, that a great and notable law marks and governs all the works of creation. It is typified in the individual mind—in our corporeal functions—in the movements of the race—and in the revolutions of the heavenly hosts—all are subject to this law of periodicity, and this alteration of condition manifests itself even in the domain of disease. We have seasons of activity and repose in the natural as in the moral world; periods of illumination and obscurity—of activity and of rest: in the one case producing day and night, winter and summer, and in the other, those alternations of social condition which have been spoken of as the Athenian age, the age of darkness, literally a long and profound intellectual eclipse, to which has succeeded the active era of mental excitement and of material progress in which we live, by which we are moved, the sun of which appears not yet to have reached its point of culmination.

In all the struggles which have marked the conflict between truth and error, ignorance and knowledge, medicine has always taken a conspicuous part, having ever been a faithful auxiliary, when not a leading element in every effort made to elevate and improve the condition of mankind, at one time allied to sacerdotal authority, as an indweller of the temples, and at another, incorporated into the body politic, or rather engrafted upon the tree of State.

In the earlier stages of civil advancement, in all those territories once composing the eastern and western empires, as is the case now in Europe, where certain forms of government exist, the sovereign authority prescribed the modes of worship, the forms of law and the requirements of medical practitioners. Unless the vigorous conservatism of these existing European governments is relaxed by the caprice of vain and foolish princes, at the suggestion of wicked men or misguided women, the right to exercise the functions of the physician is only conferred on the most satisfactory proof of indefatigable culture. And in the earlier pages of our own national history, we find the foot-prints of our European ancestors in the records of those salutary laws made for similar purposes, and transmitted to us by our political progenitors.

But in the process of time, when our form of government was changed, when the repository of sovereignty become inverted, when the power of the State passed from the few to the many, when the State became nothing and the citizens all in all, when this segregation of the sovereign power was rendered complete by the absolute freedom of the elective franchise in many of the States, then our art ceased to have a party in the commonwealth, as the law which became the exponent of this new opinion, the expression of the popular intelligence, effaced from the public record all legal traces of distinction between the physician and the hypocritical pretender.

When these ancient legal incentives to study were withdrawn, a new class of men, unprepared by mental discipline, rushed into the professional arena, bearing down by their numerical force the few remaining barriers which society was disposed to defend, notwithstanding the abrogation of law.

The political revolution which separated the American colonies from the British crown, by loosening the connection between the Church and the State, insensibly led the way to the more complete separation of medicine from governmental control and political dependence. These manifestations of popular absolutism, which swept away the prerogatives of the clerical and medical professions, threatening to involve the law in the same uncharitable equality, were the remote causes of the professional abasement we had then reached, a humiliating consciousness of which aroused its members, who in the hope of reinstating its departed dignity formed this Association.

Whatever effect this unrestricted distribution of political power through all ranks of society, may have had upon the social body, it is not our business to enquire, as ours is not a political institution, but of its immediate influence in reducing medicine to a state of degradation, there is no reason for doubting the fact nor the propriety of this exposition, for with physicians, etiology is often a key of diagnosis, and without a true pathology, there is neither safety nor certainty in the therapeutics.

In treating of medicine in its social and political relations, it is not my design, as I have no wish, even if the power were inherent, to change our organization, or to advise an essential departure from our plans of operation. I have presented the subject in this light more for the purpose of reviving the courage of members who may have begun to despair of success, because the objects we set out to accomplish, have not been at once achieved.

Time must be given for results to mature, as all social institutions are of slow growth. Those who clothe and feed the members of them, must become imbibed with a sense of their importance and necessity for their advancement, as a means of promoting the public good, else their coöperation cannot be secure. Hence our duty of endeavoring to move the social body and all its dependencies, like the horses of a Grecian Chariot all abreast, striving at the same time to shield ourselves against the propensity inseparable from the absolutism of a pure democracy, to decapitate every object that raises its head above the surrounding social level.

We have stated with philosophical accuracy, but perhaps not with strict regard to literal historic truth, that this Association was formed to repair the evils resulting from the dissevered relation of medicine to the State authority. Whatever formula we use in expressing the idea, or by whatever rationale we explain our conception of the evils said to exist, for which it was designed to furnish the remedy, the records show that its mission was to reform the medical schools of the United States, and to improve the preparatory education of students of medicine.

The development of organic bodies depends upon the absorption and assimilation of extraneous materials. If the same law regulates the growth of institutions, it becomes a matter of some interest to enquire whether the schools are an out-growth of the profession, or whether the profession is the product of the schools, for in either case, there is a labor for us to perform, and the answer to this question determines the place of beginning.

Lest a doubt might arise as to the correctness of the opinion, we wish to impress upon the professional mind, that society itself, and not we alone, are amenable to censure for the abasement to which the profession of medicine had descended at the date of our associated existence, let us for a moment look into the records of the past, to see whether we cannot find an antecedent era, in which the world has been subjected to similar moral catoclysms, by which ancient institutions were broken up, their materials converted into drift, to lay the foundations of newer and more horizontal strata, from which we may draw lessons of wisdom applicable to our own time and our own condition.

We believe that there is no period of ancient history into which that of our art is intimately interwoven, presenting more analogies to our own, and at the same time, so distinctly marked by strong antithesis, as that which intervened between the death of the Savior and Mohammed, when for more than five hundred years, a mighty struggle was going on between that Divine Word, "who lighteth every man which cometh into the world," the spirit of the Indian religion and the majesty of the Roman Empire, the latter aided by a fascinating philosophy, made beautiful by the æsthetics of Longinus, each striving for the possession of the human race.

Whilst the Empire thus labored to throw its Upas shadow over the infant church, a social disintegration of castes, owing to these struggles and the irruption of barbarian hosts from the north, took place, and a consequent universal fusion of the races, languages and customs, producing an excitation of thought, and a blending of people analogous to the social fusion and the blending of types of disease, which we see daily taking place in our own time and in our country. The minds of men thus cut loose from their ancient fastenings, sought new affinities, arranged themselves in accordance with those difficulties into new forms, many of whom wandered into unexplored paths, hoping without the aid of a Divine guide to ascertain their relation to the unseen.

Thus, also, did the members of our own profession wander into untried and forbidden paths, in pursuit of the ideal, up to the time this Association was formed. A faithful picture of the last century of this historic period, presents the deepest contrasts of light and shadow that can be portrayed in a single work of art. The darkest hue of vice being drawn in the same pannel with the purest tints of virtue. The church, young and vigorous, being soiled by its contact with a paganism inexpressibly wicked, against which it waged a war, unmitigated by acts of mercy.

These outbreaks at emancipated human thought at each of these epochs have had their use: have produced their fruits—late in arriving at maturity it is true—and especially so will it be with the germs that are scattered in the midst of the confusion of our own times. An abiding faith, that good seed, in spite of the tares that may choke it, or the birds that may devour it by the wayside, will spring up and produce fruit in good season, has led me into historical retrospect.

During the period to which we have alluded just sufficient to show what forces disdurbed and broke up ancient civilization, we find on closer examination, that the laws of the Empire relating to medicine, though unrepealed, were not enforced.

These laws made it the duty of the provincial governors to send the youth, subject to their jurisdiction, up to the city magistrates, where they were required to submit to the most rigid system of surveillance by the municipal authorities, their conduct as students, their deportment as citizens, being subjects of official scrutiny. The medical pupils under the training of the Archiatres, or State physicians, were fitted for the performance of their duties in either the wards of the cities or in towns or villages, whither they were sent by imperial authority, on the requisition of the inhabitants, who paid for their services a stipulated price.

Notwithstanding these requirements of law were left unrepealed, the new opinions which had got possession of the popular mind, being more powerful than statutes, when enforced by the Perabolani, a body of religious medical enthusiasts, and various other pretenders, who, impelled by the spirit which animates a people having just been taught to exercise the privilege of judging in matters of faith, became presumptuous in matters of science, and by applying this newly acquired right of action to medicine, having numerical strength, they overrode the prerogatives of caste, and trampled under foot the wisdom of all preceding ages.

Whilst these conflicts of opinion were being carried on in an age that produced an Athanasius, Jerome, a Chrysostom, and an Augustine, and a system of Christian ethics which absorbed into itself all that was valuable in the philosophies of Greece and Egypt, medicine acquired celebrity from such names as Caesarius, who became an Archiator—Palatinus, Oribasius, whose works remain as monuments of his genius and proofs of his culture; Actius, Alexander of Tralles, and Paulus Eguineta, scarcely inferior in reputation to the father of medicine himself. The lustre of these names seems but the more expressively to mark the twilight of that night destined thence to brood over Europe, whilst the materials of ancient civilization, broken into fragments by a rude and vigorous barbarism, were slowly wearing away the characteristics of the conquering hordes, and preparing the way for its re-appearance in new forms, through the instrumentality of the Free Cities and the feudal institutions of Europe.

During this general eclipse of letters, its occultation continuing till the art of printing was invented, we have had furnished to us an opportunity of seeing how inadequate statues alone are to the development of institutions, and how impotent they are, even when aided by professional co-operation, to resist the obstacles interposed by an adverse public opinion.

If our design has been accomplished, we have shown that the work of medical regeneration is to be commenced by the profession, whose success is made dependent upon an intelligent concurrence of the popular judgment. But it must be remembered, that in attempting to bring about essential changes in social life, in public policy, or in the constitutional relations of the different States, by whose happy form of union we are permitted to meet here to-day as fellow-citizens of a common country, we must keep in mind this fact, that all organic nature is developed from embryonic existences—that all great changes of opinion have had their origin in germs, planted long antecedent to the production of fruit, and that advances in science and improvements in the method of its application to art, have also had their seed time, their period of growth, and must ever have their day of fruition.

History is filled with exemplifications of the truth of this remark, and of evidences of the perpetuity of this law. The first step on the road to the trans-Atlantic Telegraph was taken by Volta, when he constructed the Voltaic pile—the next was the formation of the Galvanic Battery. These inventions were followed by the discovery that soft iron becomes a magnet when subjected to the action of an electrical current and resumed its normal condition as soon as the current was withdrawn. Then it was proved that the magnetic action of a current of electricity is not lengthened in intensity by passing through a long wire. Out of these antecedents, by the help of Grove's permanent battery, the Magnetic Telegraph was developed and the art of magnetic printing evolved.

But for the researches of Vesalius, who had traced out the course of the lacteals, the splendid discovery of Harvey of the circulation of the blood might have been a long time postponed.

Notwithstanding the perennial influence of those causes to which we have ascribed the tendency to professional abasement, we have met here to arrest and to counteract; there is in the condition of things by which we are surrounded, much to inspire us with confidence and to stimulate us to exertion.

We have not now, as did those who lived in the time of our historic analogue, to resist the pressure caused by the debris of an effete culture.

We have not to contend against the influence of those monstrous forms of superstition which grew out of the conjunction of Christianity, when defiled by a copartnership with the civil power, and the decaying institution of its Pagan predecessor, when a phase of credulity was developed which would prescribe the contents of a mummy case, in preference to the kreasote involved in the process of the manufacture of mummy, once an article of Egyptian commmerce.

Those political causes to which we have alluded as tending to diminish the distance between our pre-existing social extremes, whereby the medical profession lost its claim to legislative protection, have already produced the signs of a growing national homogenity, by fusing and re-casting into an American mould the various elements of which the nation is composed.

Among these materials, so readily amalgamated, which by their youth, energy and plasticity, give us our national character and national manners, there are some which need to be brought under the hammer of the forge, as well as the heat of the furnace, before they can be welded into the social mass. I allude to a class of men, wearing ecclesiastical habiliments, not wise enough to comprehend that the professions are the growths of civilization, developed by the wants and necessities of society, each one having its part to act in the drama of life, nor possessing that degree of self-respect, which would prompt a man, not even claiming to be divinely called, to avoid the contact of things proclaimed to be unclean.

When I speak of this class I do not mean the great body of American clergy—men who, instead of practicing a heterodox medicine, both practice and preach the precepts of their Divine Master as things which appertain to man's social and everlasting peace. But I mean a class, who, as if bitten by some moral Tarantula, become blinded by a phrenitic distemper, and like the great adversary of the Philistines, are ready to pull down the pillars of the temple, regardless of the ruin impending, whether that ruin involve a simple social element or the integrity of the national fabric.

Having, then, in our favor the vigor and impressibility of a new people, the resources of a new and rapidly developed country, the intelligence of a self-governing population, the augmentations of that intelligence by the unrestricted importation of learned works and the immigration of cultivated strangers, and the propulsion derived from a free and active press, we have a right to expect success. With such auxilaries, by a persistent assiduity on our part, we shall at some future day enjoy the happiness of seeing our labors crowned with the pageant of brilliant triumph.

The one thing already achieved, in the adoption and enforcement of the code of medical ethics, is worthy of commemoration by the observance of an annual holiday. Till then we had suffered more from quackery within the profession than from irregularities without. Now that order of things is reversed.

From a survey, even of the surface of society, we learn how soon the knowledge derived from medical sources strikes its roots into the popular soil. Take as example the subject of organic chemistry, and we shall see how rapidly its principles are passing into the stock of general intelligence. The numbers are daily on the increase, in every community, of those persons who know the necessity of nitrogenous articles of diet when repairs are to be made in the fibrous and areolar tissue, and how important an agency the carbo hydrogenous are supposed to exert, by increasing combustion in the removal of certain morbid conditions of the lungs.—In this way the mutual relations of the profession to the people is made apparent. The instruction communicated by the scientific physician is refunded to him in the increased capacity of the people to appreciate his worth.

We have spoken of the professions as the products of a general culture, to which, in our country, they must of necessity bear a fixed and definite relation, and of the reciprocal influences they and the society out of which they spring, exert upon each other. We have shown in a single example by what apparently simple quotations in scienti9c discovery, men are led to great practical results. As an incentive to industry, and as a reason for confidence in slow but certain success, we will detain you one moment longer, in a hosty sketch of the materials for thought that arise out of the contemplation of the field of nature, such a scene being as suggestive of thought and as full of instruction, as the examples furnished by the achievements of art.

In adjusting our telescope, to study the features of some snow-clad mountain, the organ of vision perhaps takes in the form of an enterprising explorer, whose feet still sparkling with ice as he descends from its summit, will crush out the fragrance of the plants which spring up to greet him as he walks downwards into the valley of flowers. From the eminence attained by his enterprise he could trace the course, and measure the elevation of the mountain chain, which give origin and direction to the rivers, effect the commerce, the languages and migrations of men, fix the character of the vegetation, the abode of its mammalia, and the habits of its population.

Subsidiary to the interest excited by this scene as a landscape, but not subordinate in importance, lies the geographical formation of the ranges which contain their mineral productions, give character to their fountains and increase to the variety and beauty of the vegetation, both on the slope of the mountains and in the valleys below.

Although a scene like this may excite emotion in the bosom of a savage, and awaken a sentiment of adoration for the majesty and power which can give such grandeur to nature, and even pass from the mind of an ordinary observer without any other expenditure of thought, yet to master it as a subject of scientific study would require a preparatory knowledge of what is contained in the writings of Werner and Hutton and Miller on

Geology, of Cuvier and Buckland on Paleontology, of Geoffrey, St. Hilliare and Agassiz on the races of men and the migration of Animals, of M. Balbi on Ethnography, and of Linnæus and De Candolle and Torrey and Gray on Botany. And to condense the whole into the congress of the "Cosmos" would require the genius and longevity of a Humboldt.

What is there then, gentlemen, left for us to do but to declare the perpetuity of this Association, and renew our vows of fidelity to the requirements of its constitution ?

In this proclamation and in these vows are involved the pledges, that in our professional acts we will honor the principles of moral law, which lie at the foundation of our code of Medical Ethics. That we will use our individual influence, and so try to direct the power of this Association, as to secure a higher mental culture to medical students and candidates for medical honors. When this is accomplished the medical schools will rise in character as a correlative effect, and the profession establish for itself a legitimate claim to public confidence and popular esteem. Our custom of meeting in each successive year, in a different State of the Union, prevents the decay of the body, by the introduction of new material ; and we illustrate in this way the doctrine of Zymosis, by the rapid assimilation of these new elements into the common mass. Another custom of the Association has done much to bind it to the individual States, that of shedding its honors upon the profession of the State in which the meetings are held, through which we hope to secure the sympathies of the people, and enlist them as allies in the warfore we are engaged in, against the hosts of ignorance.

A departure from the established usage of the Association, in either of these particulars, would mark the date of its decline both in vital force and mental vigor. Any restriction put upon its freedom of motion, or attempt made to centralize its influence, would enstamp it with the seal of decay.

But if the avenues to material success are so direct and brilliant, that the talent of the country is tempted to take the shorter road to wealth, whereby we fail in our attemps to lay the foundation of a national medical literature, in holding up a high exemplar to the medical student, by teaching him the necessity of a thorough preparatory discpiline before commencing professional studies, and urging him by the force of opinion, to master the elements of his profession before assuming the responsiblities which attach to the discharge of its duties, we may yet in one way leave our traces upon the national character and our foot prints on the national history, in the hallowing of one day in our annual callender, on the recurrence of which, we may have, by the example of our patriotism, the stamp of nationality, in bringing to our shrine no sectional passions, and so conducting our proceedings that brilliant memories shall adorn our annals, the names of our celebrities be embalmed as national benefactors, and the anniversaries of this Association, in honor of their services, shall form by popular consent, one of the holidays of this glorious Republic.

Often in the crisis of sectional commotion the moral necessity of a common shrine, a national feast, a place, a time, or a memory sacred to fraternal sympathies and general observance, appals the patriotic heart with regret, or warms it with desire ! Were such a nucleus for popular enthusiasm, such a goal for a nation's pilgrimage, such a day for reciprocal gratulation our own—a time when the oath of fealty could be renewed at the same altar, the voice of encouragement be echoed from every section of the confederacy, the memory of what has been, the appreciation of what is, and the hope of what may be, simultaneously felt, what a bond of union, a motive of forbearance, and pledge of nationality would be secured!

By the blessing of the Divine Founder of our holy religion, who, nineteen hundred years ago, went up to Jerusalem with his disciples to celebrate a national feast, may the proceedings of this body be so overruled, that the recollections of this meeting at Nashville to-day, when softened by the "moonlight of memory," may become a hallowed event in the annals of our yearly migration.

On motion of Dr. Flint, of Ky., the thanks of the Association were tendered to the President for his very able address, and the same was referred to the Committee on Publication.

The chairman of the Committee of Arrangements announced that the sessions of the Association would be from 9 A. M. to 2 P. M.

Judge Catron, of the U. S. Supreme Court, being present, was invited to a seat on the stand.

The Nominating Committee then retired for the purpose of nominating officers for the ensuing year

The report of the Committee on Publication being called for it was read bv Dr. Casper Wister, of Pennsylvania, and on motion, was accepted and referred to the committee on publication.

Dr. Wister also read his report as Treasurer, which was received and adopted.

On motion of Dr. Flint, of Ky., Dr. R. T. Fleming, of Ky., was admitted as a member of the Association by invitation.

The committee on Prize Essays being called upon to report, requested further time, because of the late hour at which the essays were handed in, which was granted.

The President informed the Association that Dr. F. Campbell Stewart, of New York, Dr. Alden March, of New York, Dr. Isador Gluck, of New York, and Dr. Pancoast, of Penn., had been appointed to represent this Association in foreign scientific bodies.

The committee on Medical Education was called but made no report.*

The committee on Medical Literature was called—no report.

The committee on Medical Typography and Epidemics being called, a communication from Dr. J. C. Watson, of Maine, was read, asking for further time to make a report, which was granted.

Dr. Arnold, of Georgia, offered the following resolution, which was adopted:

*Resolved*, That the committee on Nominations be constituted a standing committee during the present session of the Association, to which shall be referred all business of the Association on which an immediate vote is not required.

Dr. Jas. Mauran, of the committee on Medical Topography and Epidemics for Rhode Island, being called for, the Secretary read his apology, which was accepted.

Dr. Peregrine Wroth, of same committee for Maryland, sent in his report, with accompanying reports of Drs. A. M. White and Edmund E. Waters, which was received and referred to the committee on Publications.

---

* Received after the adjournment.

Dr. W. L. Sutton, of same committee for Kentucky, sent an apology and asked for further time, which was granted.

The members of the same committee for the States of New Hampshire, Vermont, Massachusetts, New York, New Jersey, Pennsylvania, Delaware, Virginia, District of Columbia, South Carolina, North Carolina, Tennessee and Minnesota being called, no reports were made.

The delegates from Connecticut and Louisiana being absent for the time, the considering of their reports was postponed until to-morrow.

A report from Dr. J. F. Posey, of Georgia, was presented by Dr. Arnold, and subsequently withdrawn by him for the purpose of preparing an abstract of it.

The committee on Nominations then appeared, and through their chairman, Dr. J. C. Lindsley, reported the following officers of the Association for the ensuing year, viz:

*President.*—Dr. Paul F. Eve, of Tennessee.

*Vice Presiden s.*—R. J. Breckinridge, of Kentucky, D. M. Reese, of New York, W. H. Byford, of Indiana, and Henry F. Campbell, of Georgia.

On motion of Dr. Arnold, of Georgia, the report was accepted.

The chairman stated that the Secretaries will be selected when it is ascertained where the next meeting of the Association will be held.

Dr. Wister, of Pennsylvania, moved that a committee of three be appointed by the President to conduct the newly elected officers to the chair, which was carried.

The President appointed as such committee, Drs. Wister, Arnold and McGugin.

The President elect being absent, the Association adjourned to meet at 9 o'clock, A. M., to-morrow.

---

SECOND DAY.

Nashville, May 6, 1857.

The Association met pursuant to adjournment. The minutes of yesterday were read and adopted.

The committee appointed on yesterday, Drs. Wister, Arnold and McGugin, were then requested to conduct the newly elected officers to their respective seats.

Dr. Eve, of Tennessee, in taking the chair, addressed the Association in a few pertinent remarks, as follows:

*Gentlemen of the American Medical Association:*—It is with deep emotion that I attempt to return you my heart-felt thanks for this distinguished honor. In elevating one so unworthy of this station, so ill-prepared to preside over your deliberations, or carry out the great designs of this body, I must express the apprehension that you have done yourselves injustice, and, it may be, not advanced its best interests. But, believing that this office should neither be sought nor declined, when tendered as it has been, after my State had declined to take any part in the nomination of a presiding officer, I enter upon the discharge of its onerous duties with much diffidence, and shall have frequent occasion to throw myself upon your considerate indulgence.

We are engaged, gentlemen, in a good and noble work. Life, the greatest of human blessings, and health, the sweetest stimulus of earthly enjoyments, are our end and aim. We live to secure the one and to preserve the other. To promote these all important objects, the medical profession of our country have, during the past twelve years annually appointed delegates to assemble and counsel how this may be effected. And we are here to-day on one of these great festive occasions, and, amidst our mutual congratulations, these glorious re-unions of good-will and fellowship among the brotherhood, must not forget that to us is committed the health and lives of others. In maintaining the honor and increasing the usefulness of medical science, we become the best contributors to the welfare and happiness of those around us. You have come up hither from the North and from the South, from the East and from the West, and have done well neither to count the cost nor calculate the sacrifice ; for the cause in which you are engaged is worthy of you. You present again the sublime spectacle of brethren from all sections of this widely extended Union, congregated to devise the best means to relieve suffering humanity ; and may I not add, we are here with

"Our souls by love together knit,
Cemented, mixed in one ;
One hope, one heart, one mind, one voice."

Dr. Winston, of Tennessee, read the names of additional delegates to the Association.

Dr. Hooker. from the committee on Medical Topography and Epidemics for the State of Connecticut, being called on for his report, arose and explained that it was his understanding that the committee were to have three years in which to make their report, and at the end of that time he would either be prepared or ask the indulgence of the Association for further time.

The President, under a resolution passed at the last meeting, appointed Drs. Currey, Grant and Evans, a committee on Voluntary Contributions.

Reports now being in order, the report of Dr. Posey of Georgia, was called for ; Dr. Arnold, of Georgia, read an abstract of the report of Dr. Posey; all of which, on motion of Dr. Palmer, of Michigan, was referred to the committee on Publication, under a suspension of the rule.

On motion of Dr. Wood, of New York, the reports which were presented yesterday were also referred to the committee on Publication, under a suspension of the rule.

The State of Ohio being called upon for a report upon its Medical Topography and Epidemics, the Secretary read an apology from Dr. G. Mendenhall, who asked further time in which to make a report, which was granted.

The States of Mississippi, Missouri, Michigan, Illinois, Indiana, Wisconsin, Iowa, California, and the U. S. Navy, being called, no response was made.

A telegraphic despatch from Dr. J. M. Sims, of New York, who was to report on the Treatment of the Results of Obstructed Labor, was received and referred to the appropriate committee.

A communication was received from the Southern Methodist Publishing House, inviting the members of the Association to visit that establishment, which was accepted.

A communication was read by Dr. Lindsley, of Tennessee, from the Medical Association of Washington City, inviting the National Association to hold their next annual meeting in that city. On motion the communication was referred to the committee on Nominations.

A resolution was offered by Dr. Bartlett of Wisconsin, tendering a vote of thanks to the late President, Zina Pitcher, for the able manner in which he has presided over the deliberations of this body, which was unanimously adopted.

The reports of Special Committees for 1856–7, being next in order, they were called in order as follows:

*Inflammation—its Pathology, etc.*—Dr. E. R. Peaslee, Maine; asked further time. Referred.

*Anatomy and Histology, of the Cervix Uteri.*—Drs. H. Hutchinson and Charles E. Isaacs, New York; no report.

*Treatment of Cholera.*—Dr. J. Taylor Bradford, Kentucky; no report.

*Treatment best adapted to each variety of Cataract, etc.*—Dr Mark Stephenson, New York; further time asked. Referred.

*Causes of the Impulse of the Heart, etc.*—Dr. J. W. Corson, of New York; a communication was received, and on motion of Dr. Brodie, he was continued.

*Causes of Infant Mortality, etc.*—Dr. D. Meredith Reese, of New York, read an abstract of his report, which was referred to the committee on Publication.

The venerable Dr. Shelby, of Tennessee, being present, was invited to a seat on the stand. His appearance was warmly acknowledged.

Dr. Hobbs, of Illinois, offered the following resolution:

*Resolved*, That a committee on Essays, (not including Prize Essays,) be appointed, to whom all essays prepared for publication by this Association shall be referred, which committee shall transfer to the committee on Publication, all Essays they judge worth publishing. That said committee on essays, make a full report of their proceedings to the Association at its next annual session; provided, authors of rejected essays being informed of said rejection by said committee, shall have the privilege of withdrawing their essays from the report of the committee to the Association.

On motion of Dr. Palmer of Michigan, the resolution was indefinitely postponed.

Thh Secretary read a protest signed by Drs. Richard Arnold, J. Gordon Howard, Pike Brown and George P. Pandleford, against admitting the delegates from Oglethorpe Medical College, as follows:

NASHVILLE, May 6, 1857.

The undersigned, members of the American Medical Association, protest against the admission of delegates from the Oglethorpe Medical College of Savannah, on the ground that it is not a regularly organized college, it being a matter of public notoriety in Savannah, that during neither of the two sessions of its existence, have all the chairs been regularly filled. During its first session the chairs of Physiology and Materia Medica were not filled, except by a very few lectures, by the gentleman appointed to them, and the same thing occurred during its last session as to the Chairs of Materia Medica and Chemistry. All of which is respectfully submitted.

RICHARD D. ARNOLD, M. D.

J. GORDON HOWARD, M. D.

PIKE BROWN, M. D.

GEO. P. PADLEFORD, M. D.

After several resolutions were offered and some discussion,

On motion of Dr. Palmer, the whole subject was referred to a committee of three to be appointed by the chair.

Dr. Brodie of Michigan, moved as an amendment, that no Faculty Member of a Medical College be appointed upon the committee, which was accepted by the mover.

The Chair appointed as such committee, Drs. Wister, of Pennsylvania, Bemiss, of Kentucky, and Gibbs, of South Carolina.

Dr. Felix Robertson, the oldest physician in Tennessee, being present, was invited to a seat on the stand. He was greeted with marked consideration by the Association.

The Committee on Nominations was convened to transact important business.

The calling of Special Committees was resumed:

*Spontaneous Umbilical Hemorrhage, etc.*—Dr. J. Foster Jenkins, New York. Further time asked. Referred.

*Use of Instruments in Obstetrical Practice.*—Dr. Henry Carpenter, of Pennsylvania. No report.

*Measures to be adopted to Remedy the Evils existing in the present mode of holding Coroner's Inquests.*—Dr. Alexander J. Semmes, D. C. Report presented with the following resolution attached:

*Resolved*, That committees of three, in each State, Territory and the District of Columbia, be appointed, and that said committee be, and they are hereby authorized in the name of this Association, to memorialize their respective Legislatures, to pass such laws as will best carry into effect the objects of the foregoing report.

The report was referred to the Committee on Publications, and the accompanying report adopted and referred to the committee on Nominations.

*True Position and Value of Operative Surgery, etc.*—Dr. J. B. Flint, of Kentucky. Further time asked; granted.

*Causes and Cure of Indigestion, etc.*—Dr. G. Volney Dorsey, of Ohio. No report.

*Medical Jurisprudence of Insanity, etc.*—Dr. C. B. Coventry, of New York. Further time granted.

*Human, Animal and Vegetable Parasites.*—Dr. Joseph Leidy, Pennsylvania. No report.

*Value of strict attention to position in the Treatment of Diseases of the Abdomen.*—Dr. M. D. Darnall, Indiana. No report.

*Milk Sickness.*—Dr. George Sutton, Indiana. No report.

*Blending and Conversion of the Types of Fever.*—Dr. Clark G. Pease, Wisconsin. Communication sent, but not received Postponed.

*Best Substitutes of Cinchona, etc.*—Dr. B. S. Woodworth, Indiana. No report.

*Use of Cinchona in Malarious Diseases.*—Dr. Franklin Hinkle, Pennsylvania. Report furnished. Referred to Committee on Publication.

*Nervous System in Febrile Disease.*—Dr. Henry F. Campbell, Georgia. Verbal abstract of report given. Referred to Committee on Publication.

*Laws Governing the absorption and Deposit of Bone.*—Dr. John Neill, Pennsylvania. No report.

*Intimate Effects of Certain Toxicological Agents in the Animal Tissues and Fluids.*—Dr. John W. Green, New York. No report.

*Intimate Structure and Pathology of the Kidney.*—Dr. Charles E. Isaacs, New York. Further time granted.

*Diseases Incidental to Emigrants, etc.*—Dr. Israel Moses, New York. No report.

*Etiology and Pathology of Epidemic Cholera.*—Dr. T. W. Gordon, Ohio. Partial report presented and referred.

*Excretions as an Index to the Changes going on in the System.*—Dr. H. A. Johnson, Illinois. No report.

*Remedial Effects of Chloroform.*—Dr. D. D. Thompson, Kentucky. No report.

*Best Method of Causing an Increase in the number of Essays, etc.*—Committee: Drs. Leidy, Wood and Meigs, Pennsylvania. No report. Committee continued.

*Changes produced in Composition and Properties of Milk, etc.*—Dr. N. S. Davis, Illinois. Communication read and further time granted.

*Stomatitis Materna.*—Dr. McGugin, Iowa. Further time granted.

An abstract of the report of Dr. Fenner, of Louisiana, upon the Medical Topography of that State, was then read and referred.

Dr. Singleton, of Kentucky, offered the following resolution, which was unanimously adopted:

*Resolved*, That in the death of Dr. Grafton, of Mississippi, the American Medical Association has lost a talented and useful member, and society a benefactor.

On motion of Dr. Whitaker, of Tennessee, Dr. H. Ronalds was expelled from the Association for giving certificate contrary to the rules of the Association.

Dr. Caspar Wister, chairman of the committee upon the admission of the delegates from Oglethorpe Medical College, reported as follows:

Dr. W. Benson asserts that for the past session the Oglethorpe school has been fully organized, that six professorships have been regularly filled, and that the occupants of these chairs have been in the constant fulfillment of their duties, except in cases of illness; such instances having, however, at no time interrupted the regular course of lectures, the professor absent having had his place supplied by his colleagues. The seventh chair is admitted to have been vacant; the duties were discharged however, fully by other members of the faculty.

Dr. R. D. Arnold prefers no charges beyond those admitted above.

Therefore, your committee finding nothing that infringes upon the strict letter of the law of the American Medical Association, in reference to the admission of members, we recommend that all further action in this question be suspended.

CASPAR WISTER,
R. W. GIBBES,
A. M. BEMISS.

The Secretary read the following preamble and resolutions, which were unanimously adopted:

WHEREAS, It has pleased God to remove by death our fellow-member, Robert M. Porter, and because of his devotion to the interests of the Profession of Medicine, and his steady support of the American Medical Association,

*Resolved*, That this Association learned with unfeigned sorrow of his decease; and that they have lost a firm and intelligent supporter, and society a benefactor and friend.

Dr. T. Bullard, of Indiana, offered the following:

*Resolved*, That in the death of Dr. John L. Mothersett, this Association has lost a useful member, and Society a beecfactor.

The Secretary read a communication from the Connecticut Medical Society, asking that the time for holding the meetings of the Association in northern cities be changed to a later period in the year. Referred over to the next meeting by the Constitution. Adjourned to meet at 9 o'clock, A. M., to-morrow.

THIRD DAY.

NASHVILLE, May 7, 1857.

The Associatian met pursuant to adjournment. The minutes of yesterday were read and adopted.

Dr. Hoyte, from the Committee of Arrangements, read the names of additional delegates to the Association, who had arrived since the meeting of the Association yesterday.

The Secretary read a communication from Dr. Clark G. Pease, of Wisconsin, which accompanied his report on "*Blending and Conversion of the Types of Fever*"

Dr. Hooker of Connecticut, moved that the report be referred to the Committee on Voluntary Contributions.

Dr. McKinley moved to amend by having a portion of the report read, which was lost, and the motion recurring to refer the report, it was carried.

Dr. Currey, from the Committee on Voluntary Contributions, submitted the following report, which was accepted :

The Committee on Voluntary Contributions has examined the following papers, and recommend them for publication in the Transactions of the Association :

1st. A new Principle of Diagnosis in Dislocations of the Shoulder Joint. By L. A. Dugas, M. D., Professor of Surgery in the Medical College of Georgia, Augusta ; accompanied by four photographic plates illustrating the principle.

2d. Medical Statistics of Washington Territory. By George Suckley, M. D., U. S. A., embracing, 1st, Geological Divisions of the Territory; its Geology, Meteorology, Fauna. 2d, White population and its diseases. 3d, Native population ; Diseases ; Medical Practice ; causes of their rapid disappearance ; concluding remarks.

3d. Medical Flora of Washington and Oregon Territories. By J. G. Cooper, M. D.

All of which is respectfully submitted.

R. O. CURREY,
R. T. EVANS,
GEO. R. GRANT.

Dr. Yandell offered the following resolution :

*Resolved*, That this Association re-affirm the principles respecting the rights of constituent bodies announced in a report contained in Volume V., of its Transactions, in the following terms :

" The Faculty of every Medical College, shall have the privilege of sending two delegates to this Association, *provided*, that the said Faculty contain not less than six Professors, who give one course of instruction annually, of not less than six weeks, on Anatomy, Materia Medica, Theory and Practice of Medicine and of Surgery, Midwifery, and Chemistry; and also that said Faculty requires that its candidates for graduation, among other requisites, shall have attended two full courses of lectures with an interval of not less than six months between them, one of which courses must have been in their Institution.

Dr. Breckinridge in the Chair.

Dr. Buchanan proceeded to discuss the resolution, and at the close of his remarks, moved to lay it on the table, which was subsequently withdrawn.

Dr. Boring offered the following resolutions in lieu, which he proceeded to discuss:

*Resolved*, That this Association has not the power to control the subject of Medical Education.

*Resolved*, That the great objects of this Association are the advancement of Medical Science, and the promotion of harmony in the profession.

*Resolved*, That the attempt upon the part of this body to regulate Medical Education, having most signally failed in its object, and already introduced elements of discord, any further interference with this subject would not only be useless, but calculated to disturb and distract the deliberations of this Association.

Dr. Currey offered the following resolutions in lieu of the whole:

*Whereas*, The subject of Medical Education has been committed at each annual Session to Standing Committees, and various suggestions have been proposed, which the Association has adopted, and recommended to private instructors and to the Medical Colleges.

*Resolved*, That a committee of five be appointed by the Committee on nominations, as a Special Committee, to be composed of members who are in no respect connected with any Medical School, to devise a *System of Medical Instruction*, to be presented for the consideration of this Association at its annual Session in 1858.

*Resolved*, That the proposed system shall set forth a uniform basis, upon which our Medical Institutions shall be organized, as well as have reference to the best mode of securing the Preparatory Medical Instruction to the Student, and that consequently the legitimate subjects to be embraced in said system, will include Primary Medical Schools—the number of Professorships in Medical Colleges, the length and number of terms during the year, the requisite qualifications for graduation, and such other subjects of a general character as to give uniformity to our Medical system, and preserve harmony and friendly intercourse in the ranks of the profession.

*Resolved*, That upon the adoption of the proposed system by the Association, all Institutions which may conform to it shall be entitled to representation at the Annual Sessions of this Association and none others.

The subject was further discussed by several members of the Association.

Dr. Reese, after some remarks, moved the indefinite postponement of the whole subject; which was lost.

Dr. Arnold moved the previous question, which was lost, and the discussion proceeded at considerable length, when

Dr. Hooker moved the previous question on the resolutions of Dr. Currey.

The reading of the various resolutions being called for, they were read to the Association.

The motion of Dr. Hooker being in order, the previous question was called, and the resolutions of Dr. Currey were adopted.

Dr. Lindsley, from the Nominating Committee, submitted the following report:

*Secretaries.*—Robert C. Foster, of Tennessee, A. J. Semmes, of Washington City.

*Treasurer.*—Caspar Wister of Philadelphia.

For the next place of meeting, Washington City.

STANDING COMMITTEES.

*Committee of Publication.*—Francis G. Smith, of Philadelphia, chairman; Caspar Wister, of Philadelphia; R. C. Foster, of Nashville; A. J. Semmes, of Washington City; Samuel L. Hollingsworth, of Philadelphia; Samuel Lewis, of Pennsylvania; H. F. Askew, of Delaware.

*Committee on Prize Essays.*—Grafton Tyler, of Georgetown, D. C., chairman; J. C. Hall, of D. C.; J. F. May, of D. C.; Thomas Miller, of D. C.; A. J. Semmes, of D. C.; Joshua Riley, of D. C.; W. J. C. Duhamel, of D. C.

*Committee of Arrangements.*—Harvey Lindsly, chairman, W. J. C. Duhamel, Cornelius Boyle, P. H. Coolidge, G. M. Dove, A. Y. P. Garnett, Wm. P. Johnston, of D. C.

*Committee on Medical Education.*—G. W. Norris, of Philadelphia, chairman; A. H. Luce, of Illinois; E. R. Henderson, of South Carolina; G. R. Grant, of Tennessee; T. S. Powell, of Georgia.

*Committee on Medical Literature.*—A B. Palmer, of Detroit, chairman; A. F. Alexander, of Alabama; J. M. Mosgrove, of Ohio; P. Cossidy, of Pennsylvania; S. Pollak, of Missouri.

*Vacancies in Committee on Medical Topography and Epidemics.*—T. B. Shuford, to fill the vacancy caused by the death of Dr. Grafton, of Mississippi. C. W. Parsons, to fill the vacancy caused by the resignation of Joseph Mauran, of Rhode Island.

SPECIAL COMMITTEES.

*Spontaneous Umbilical Hemorrhage of the newly born.*—J. Foster Jenkins, of New York.

*Influence of Marriage of Consanguinity upon Offspring.*—Dr. Bemiss, of Kentucky.

*Functions of Different portions of the Cerebellum.*—E. Andrews, of Illinois.

*Causes of the impulse of the Heart and the agencies which influence it in health and disease.*—J. W. Corson, of New York City.

*Treatment of the Results of Obstructed Labor.*—J. Marion Sims, of New York.

*Treatment best adapted to each variety of cataract, with the method of operation, place of election, time, age, etc.*—Mark Stephenson, of New York.

*Human, Animal and Vegetable Parasites.*—Joseph Leidy, of Philadelphia.

*Best Substitutes for Cinchona and its preparation in the treatment of Intermittent Fever, etc.*—B. S. Woodward, of Indiana.

*Intimate structure and pathology of the Kidney.*—Charles E. Isaacs, of New York.

*Etiology and Pathology of Epidemic Cholera.*—T. W. Gordon, of Ohio.

*Inflammation of Cervix Uteri.*—Henry H. Miller of Louisville, Kentucky.

*On Milk Sickness.*—W. H. Byford, of Indiana.

*Best means of causing an increase of the number of Essays.*—Drs. Leidy, Wood and Meigs, of Pennsylvania.

*Changes produced in Composition and Properties of Milk.*—N. S. Davis, of Illinois.

*Stomatitis Materna.*—D. C. McGugin, of Iowa.

*On Criminal Abortion with a view to its general suppression.*—H. N. Storer, of Boston.

The committee recommend that the committees ordered by the adoption of the resolutions accompanying Dr. A. J. Semmes' report, be filled by the several State Societies.

On motion of Dr. Brodie, amended so as to refer the same to the officers of several State Societies. Carried.

The committee also recommend the amendment of the third article of the constitution, in relation to meetings, by inserting after the words "first Tuesday in May," the words, *or the first Tuesday in June*, and also by inserting after the words "shall be determined," the words, *with the time of meeting.*

*Special Committee on the present state of science, as regards the Pathology and Therapeutics of the Re-productive Organs of the Female.*—D. Fordyce Barker, of New York.

*On Moral Insanity.*—D. M. Reese, of New York.

*On Calculi and the Diseases of the Urinary Organs, in Iowa, Minnesota, and Nebraska.*—Dr. J. C. Hughes, of Keokuk, Iowa.

*On the nature, tendency and general treatment of Syphilitic Bubo.*—Moses Gunn, of Detroit, Michigan.

*Organic Chemistry—its progress and relations to Physiology and Pathology*—Professor Samuel St. John, of New York.

*On Medical Education.*—(By Dr. Currey's resolution,) James R. Wood, of New York; George R. Grant, of Memphis, Tennessee; John Watson, of New York; C. B. Nottingham of Macon, Georgia; Rene La Roche, of Philadelphia, Pennsylvania.

*To fill a vacancy in the Committee on Medical Topography and Epidemics.*—Dr. J. L. Cabell, of Charlottsville, Virginia.

Dr. March moved that the Report of the Nominating Committee be taken up, and each subject to which it refers, be considered separately, which motion prevailed. That portion relating to nominations was then adopted.

The place of the next annual meeting of the Association being the next subject in order, after some discussion, on motion of Dr. March, the report of the committee was adopted.

Dr. Lindsley moved that, as Dr. Semmes, one of the newly elected Secretaries was absent, Dr. Brodie, of Michigan, be elected Secretary *pro tem*, which was carried.

Dr. Pitcher offered the following resolution, which was unanimously adopted:

*Resolved*, That a committee of three be appointed, of which the President of the Association shall be chairman, to communicate with the

Surgeon General of the Army, the chief of the Medical Bureau of the Navy, and the Secretary of the Treasury of the United States, with a view to secure the concurrence of these departments of the Federal Government, so that its contributions to the Medical Topography, the Vital Statistics, and the Sanitary Police of the nation may be made tributary to the labors of this Association.

The Chair appointed as such committee, Drs. Z. Pitcher, of Michigan, and R. H. Coolidge, of Kansas.

Dr. Bowling, Chairman of the Committee on Prize Essays, submitted the report of said Committee, as follows:

The Committee on Prize Essays report that four essays have been received, each possessing great merit.

The Committee selected the following two Essays for the two prizes, provided for at the last meeting of this Association.

1st. One entitled "The Excreto-Secretory System of Nerves. Its relation to Physiology and Pathology," with the following motto:

"*Observation becomes Experiment when used in severe processes of Induction*," and signed Henry Fraser Campbell, Georgia.

2nd. "Experimental researches relative to the Nutrition, Value and Physiological Effects of Albumen, Starch and gum when singly and exclusively used as Food," with the following motto:

"*Qum sequimur? quove in jubes? ubi ponere sedis?*

*Da pater agurium, atque animis illabere nostris!*" and signed, William A. Hammond, M. D., Assistant Surgeon, U. S. Army.

The President read an invitation to the members of the Association, to visit the University of Nashville, in its Military, Literary and Medical Departments.

The Committee on Voluntary Contributions, reported in favor of the publication in the Transactions of the Association, of the following paper. "On the blending and conversion of the Types in Fever." By C. S. Pease, M. D., of Wisconsin. The report was adopted.

Dr. McMurray offered the following resolution which was adopted:

*Resolved*, By this Association, that the Committee on Publications be instructed to append the Code of Ethics of the American Medical Association to each volume of its present and future Annual Transactions.

The amendments to the Constitution proposed by Dr. Stocker, of Pa., at the last Annual Session, were taken up and laid on the table.

Dr. Lindsley offered the following amendment to the Constitution, which was seconded by Dr. Gunn:

"In Art. II, omit the words 'Medical Colleges' and also the words 'The Faculty of every regular constituted Medical College, or chartered School of Medicine, shall have the privilege of sending two delegates.'"

The amendment lies over until the next meeting of the Association, under a rule of the organization.

On motion of Dr. Palmer, the resolutions reported at the last Annual meeting of the Association, by the Committees on Plans of Organization for State and County Medical Societies were taken up and adopted.

The following resolutions were offered and adopted:

By Dr. Pitcher—

*Resolved*, That the members of this Association, as recipients of the cordial, generous, and elegant hospitalities extended to them by the profession and the citizens of Nashville, in placing on record an expression of thanks for the social amenities they have enjoyed during its tenth annual session, wish also to leave behind them the assurance, that the recollection of their short sojourn in Tennessee, will be cherished as dearly as the remembrance of the far off sound of water, by the exhausted and way-worn traveler.

By Dr. Means—

*Resolved*, That the earnest thanks of this body be presented to the authorities of the State and City, who have tendered this magnificent State Capitol for their sittings during the present session.

By Dr. Currey—

*Resolved*, That the thanks of the Association be tendered to the Reporters of the City Press, for the accuracy and promptness with which they have reported the proceedings of the Association, and to the Publishers, for the liberal supply of their morning papers during the Sessions of the Association.

By Dr. Wister—

*Resolved*, That the thanks of this meeting be presented to Dr. Wm. Brodie, for the efficiency with which he has discharged his duties of Secretary.

By Dr. Byford—

*Resolved*, That the State and County Societies throughout the Union be requested to recommend their members to purchase the Transactions of the American Medical Association, and that their officers act as agents for the same.

On motion of Dr. Gunn, of Michigan, the Association recognized the presentation of a pamphlet by Henry Fraser Campbell, M. D., claiming "Priority in the Discovery and naming of the Excito-Secretory System of Nerves."

On motion of Dr. Byford, the Association then adjourned *sine die*.

